# National workshop on ECT practice

**DOI:** 10.4103/0019-5545.46082

**Published:** 2005

**Authors:** Jagadisha, Nilesh Shah, Prathap Tharyan, B.N. Gangadhar

**Affiliations:** * Assistant Professor National Institute of Mental Health and Neuro Sciences, Bangalore; **** Professor National Institute of Mental Health and Neuro Sciences, Bangalore; *** Professor Christian Medical College, Vellore; ** Professor, Lokmanya Tilak Memorial Medical College, Mumbai

Dear Editor,

In 1990, a national workshop on the practice of electroconvulsive therapy (ECT) was held at NIMHANS, Bangalore. Practice guidelines for ECT were formulated at that time.^1^ In the ensuing 15 years, several advances were made in the field of ECT. Many of these advance were discussed at the ECT certificate course at Annual National Conference of Indian Psychiatric Society (ANCIPS)-2005, held in Chandigarh. We report a few of our observations and views about the course.

The workshop was open to members of the Indian Psychiatric Society—both practising and trainee psychiatrists. As this event was conceived as a certificate course, the number of registrations was restricted to 100 on a first-come, first-served basis. The response to the announcement of this workshop was overwhelming; 244 psychiatrists applied for registration. Of the 100 who had confirmed registration, 91 attended the workshop. The course consisted of presentations by experts on three major topics—(i) consent and anesthetic standards; (ii) stimulus and seizure monitoring; and (iii) concurrent drugs and special populations. Each presentation was followed by a discussion. A panel discussion was conducted to summarize the course.

We evaluated the certificate course in two ways. First, the participants were asked to rate two aspects of the presentations—content and clarity—on a scale of 0 (very bad) to 10 (very good). Seventy-three participants (80%) returned the evaluation form. They rated both the content and the clarity of the presentations highly; question-and-answer sessions, panel discussions and the overall learning value of the course were also rated highly (Table 1).

**Table 1:** Evaluation of the certificate course by participants on a scale of 0 to 10 (*n*=73)

	Minimum	Maximum	Mean	SD
Consent and anaesthetic standards				
Content	5	10	8.12	1.47
Clarity and presentation	6	10	8.67	1.12
Stimulus and seizure monitoring				
Content	4	10	8.25	1.49
Clarity and presentation	5	10	8.40	1.37
Concurrent drugs and special populations				
Content	5	10	8.47	1.25
Clarity and presentation	6	10	8.70	1.16
Learning new information	3	10	8.22	1.61
Questions and answers and panel discussion	5	10	8.10	1.45

Second, we evaluated the knowledge of the participants at the end of the course by means of a multiple-choice questionnaire. There were 35 questions on the basic and practical issues of ECT. Fifty participants (55%) returned the answers. The mean score was 23.16 (±6.24), which was 65% of the maximum score. The maximum scores were obtained in the indication subsection (82%) and the minimum in the seizure monitoring subsection (54%). The scores in other subsections including contraindications, complications, concomitant medication, anaesthesia and stimulus parameters were in the range of 64%–69%. Eighty-two per cent of the respondents got more than 50% of the maximum score.

We asked the respondents whether there was a need for conducting a national workshop on ECT. Ninety-two per cent thought that it was a good idea; only 2 respondents (2.7%) thought otherwise. Finally, we asked the respondents for their comments and suggestions. Many respondents suggested that a workshop with emphasis on practical issues and live/video demonstration of ECT procedures should be conducted.

The overwhelming response to the certificate course, the fact that about 75% of aspirants could not be accommodated, and the feedback gained from the participants indicates the necessity to conduct another national-level workshop on the practice of ECT. We strongly recommend that the Indian Psychiatric Society consider conducting such a workshop. The developments in the area of ECT call for practice guidelines on ECT—a position document of the Indian Psychiatric Society.
